# Supplementary figures and images for: Proteolysis of fibrillin-2 microfibrils is essential for normal skeletal development
Source: eLife. 2022 May 3;11:e71142. doi: 10.7554/eLife.71142 (PMC9064305; doi:10.7554/eLife.71142)

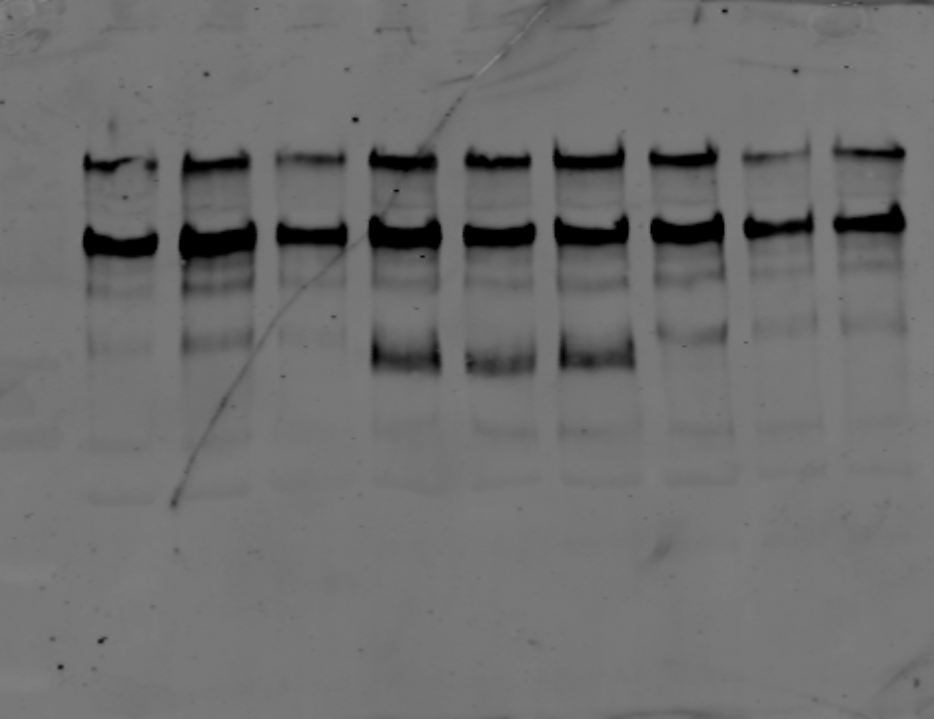

Supplement: Source data 1. [file elife-71142-data1.zip › eLife source data/Figure 7-source data 1.tif]

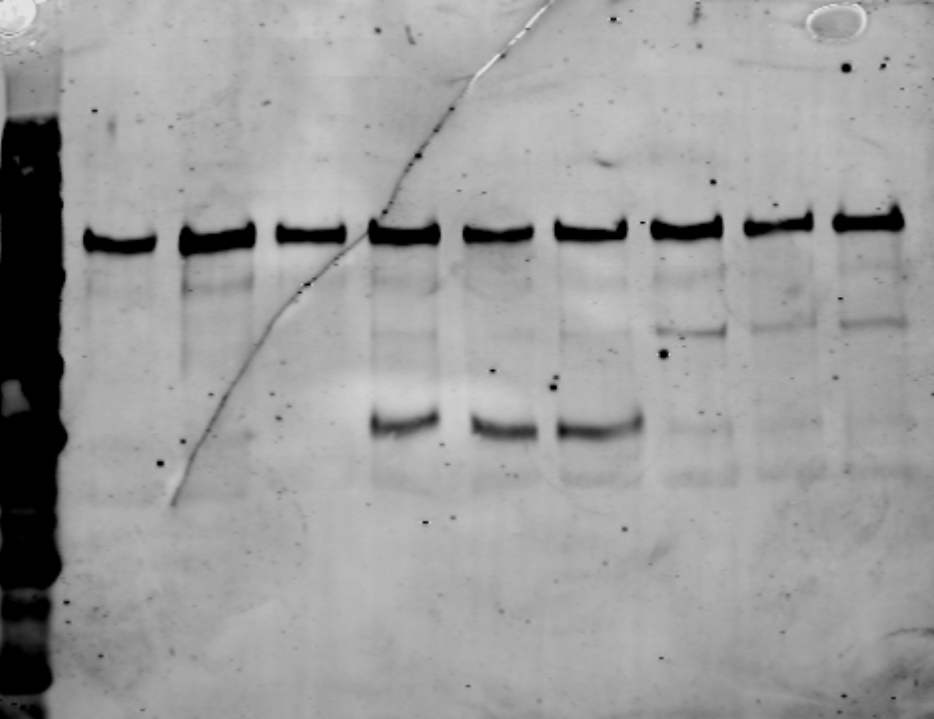

Supplement: Source data 1. [file elife-71142-data1.zip › eLife source data/Figure 7-source data 2.tif]

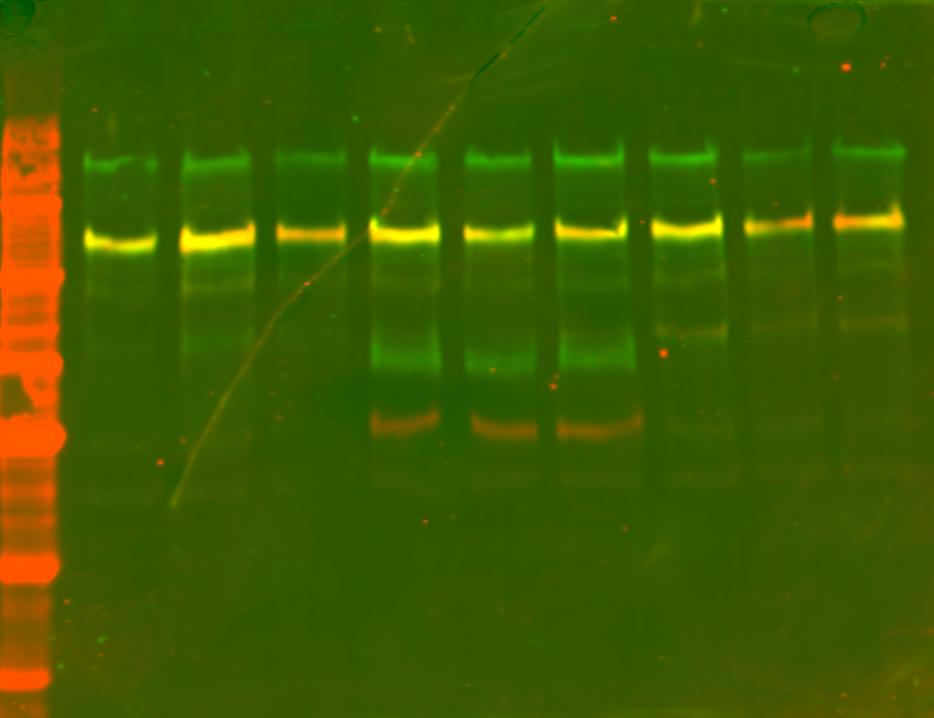

Supplement: Source data 1. [file elife-71142-data1.zip › eLife source data/Figure 7-source data 3.tif]

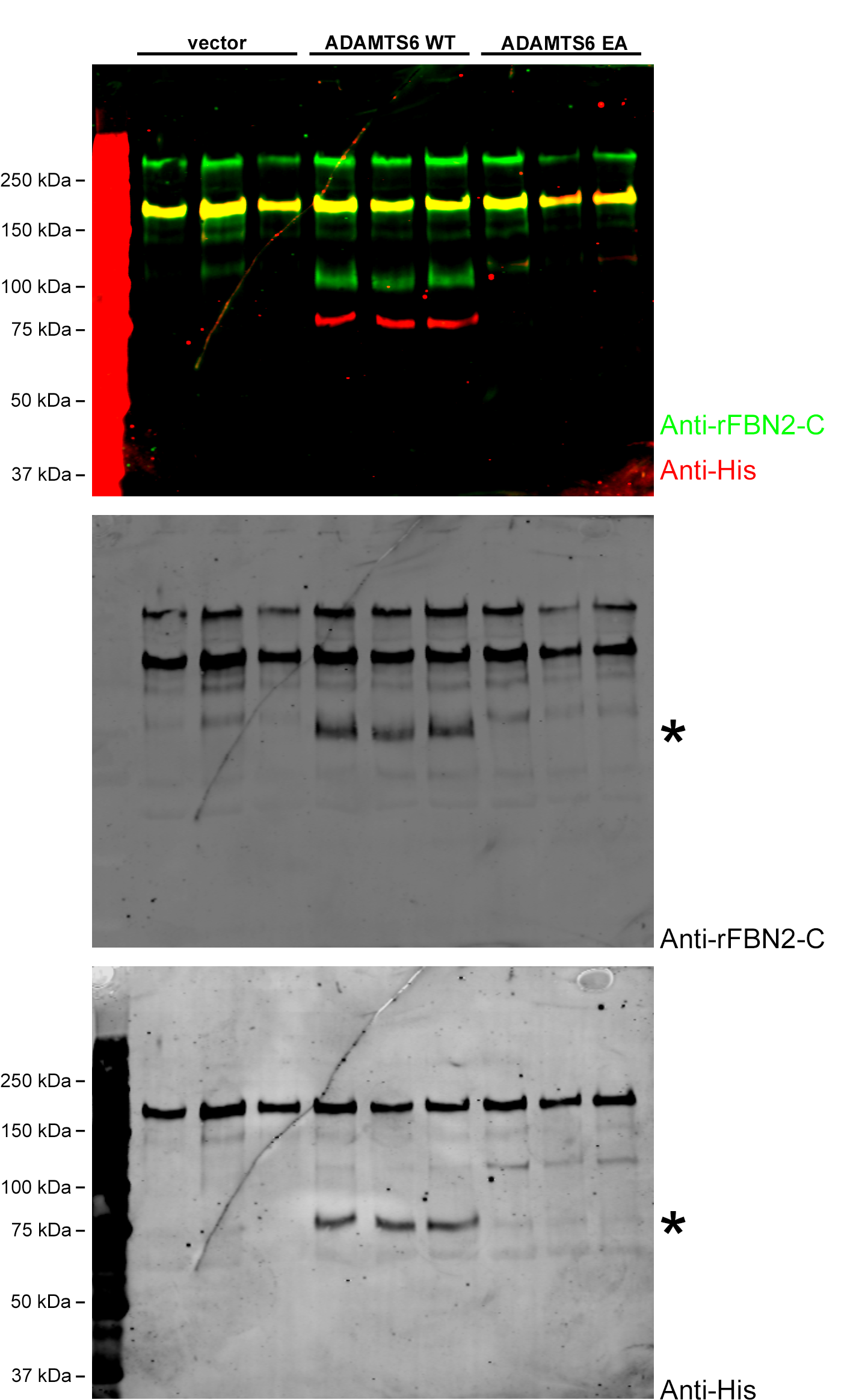

Supplement: Source data 1. [file elife-71142-data1.zip › eLife source data/Figure 7-source data 4.tif]

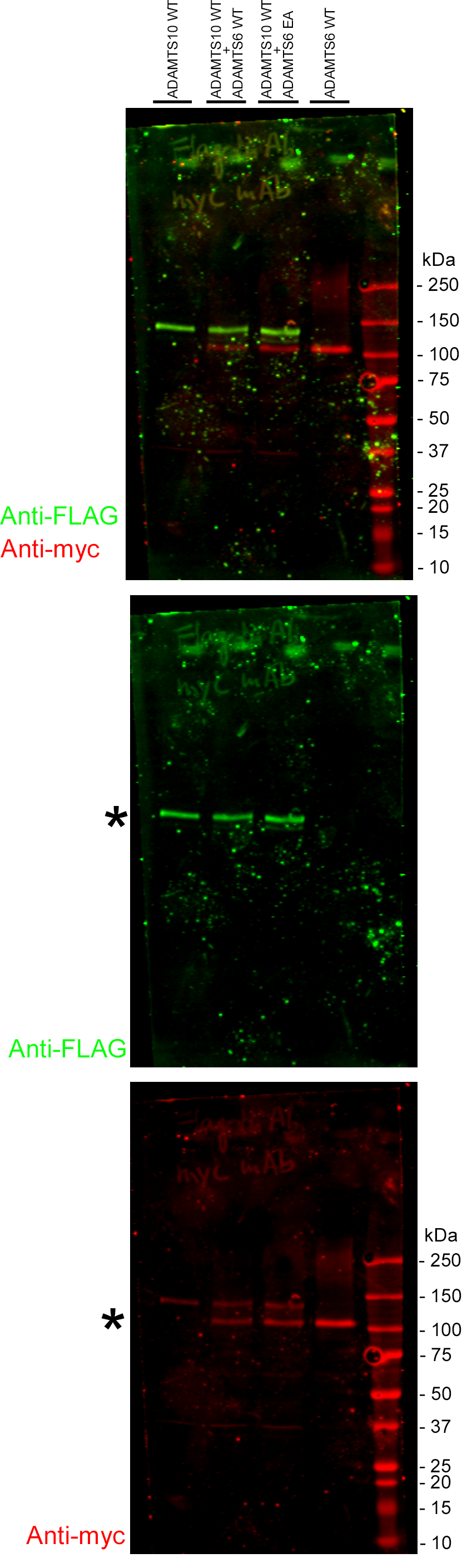

Supplement: Source data 1. [file elife-71142-data1.zip › eLife source data/Figure 1-source data 4.tif]

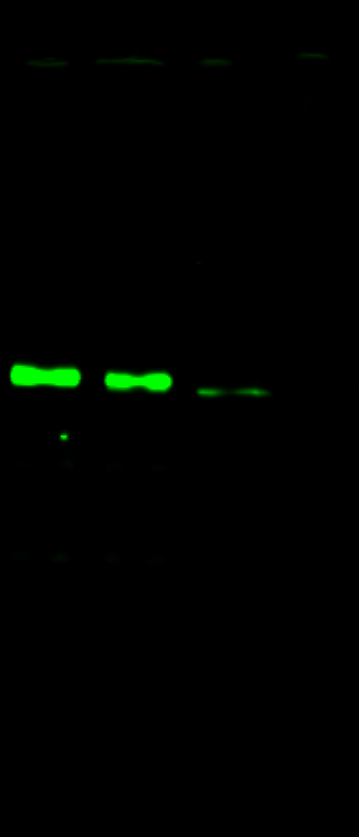

Supplement: Source data 1. [file elife-71142-data1.zip › eLife source data/Figure 1-source data 5.tif]

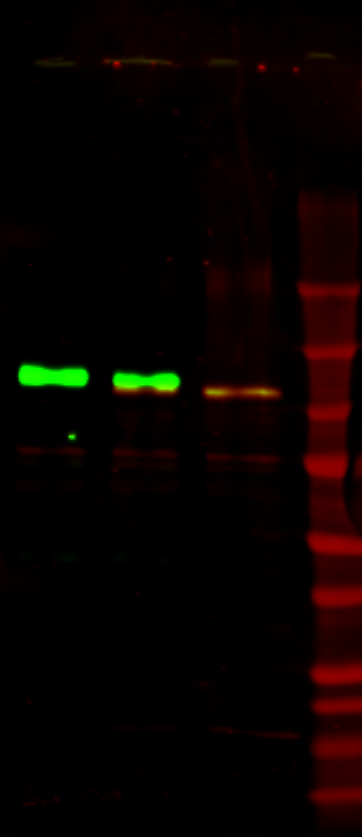

Supplement: Source data 1. [file elife-71142-data1.zip › eLife source data/Figure 1-source data 7.tif]

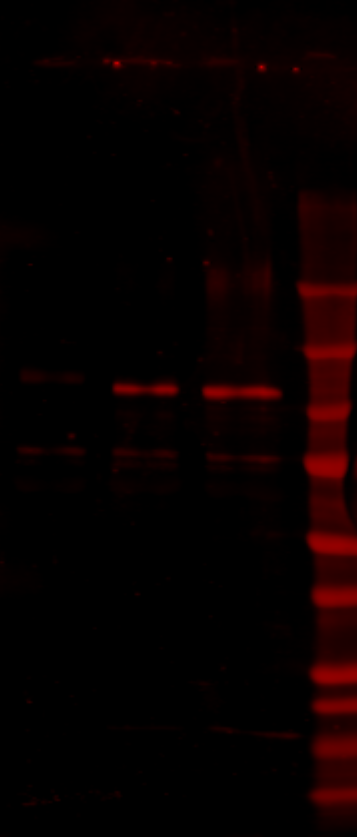

Supplement: Source data 1. [file elife-71142-data1.zip › eLife source data/Figure 1-source data 6.tif]

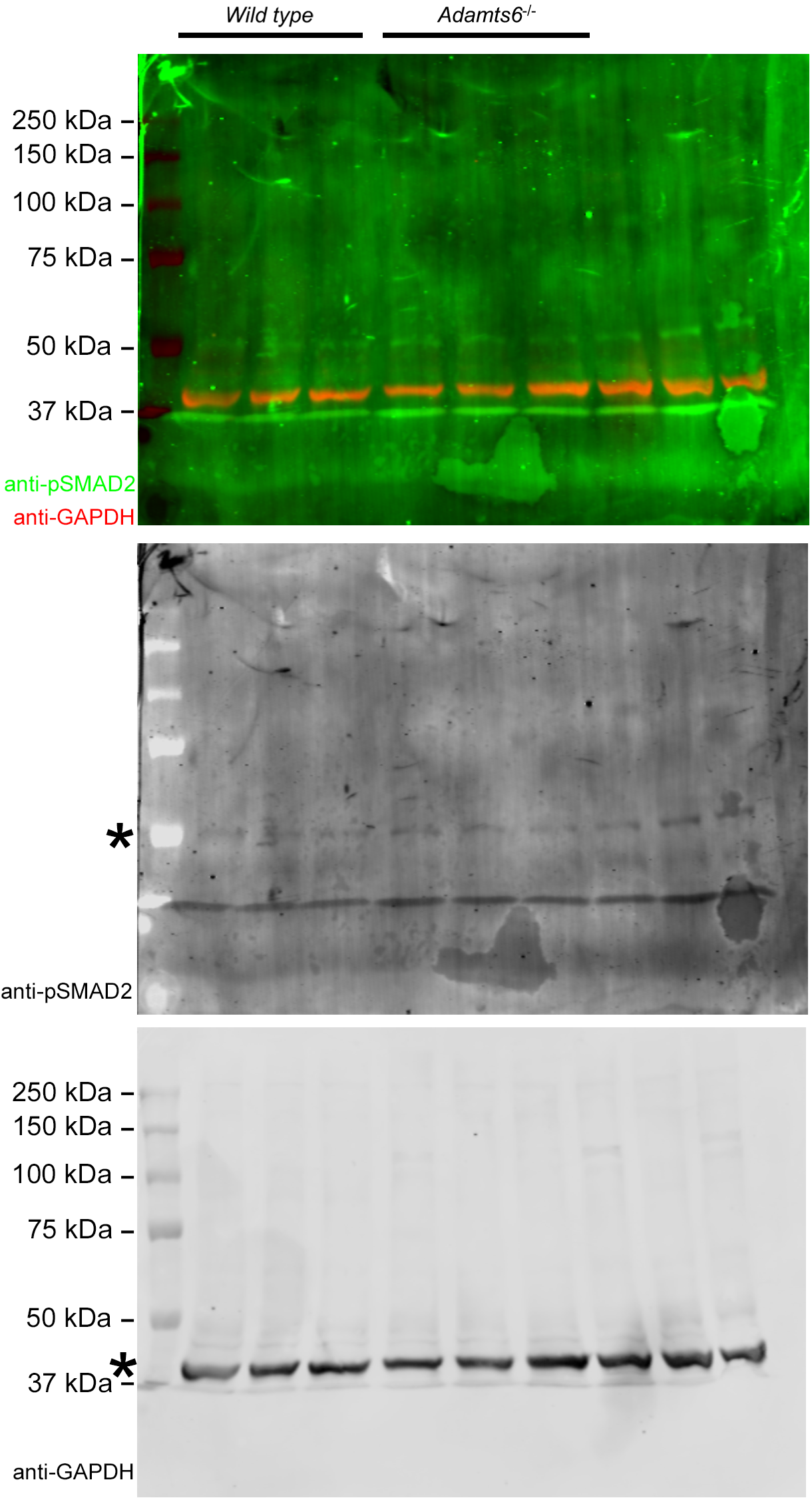

Supplement: Source data 1. [file elife-71142-data1.zip › eLife source data/Figure 9-source data 8.tif]

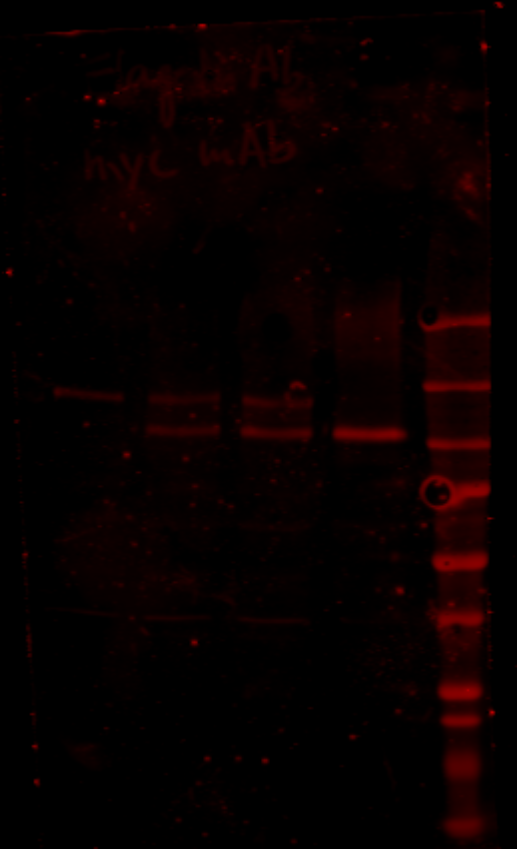

Supplement: Source data 1. [file elife-71142-data1.zip › eLife source data/Figure 1-source data 2.tif]

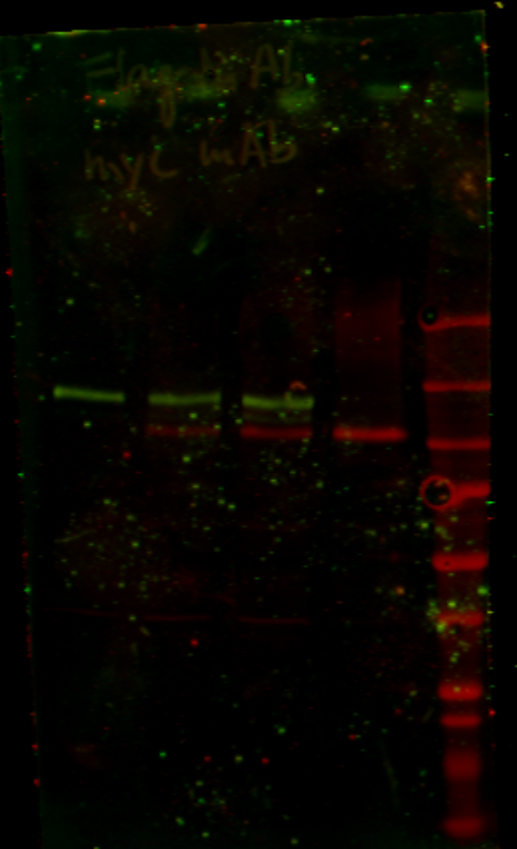

Supplement: Source data 1. [file elife-71142-data1.zip › eLife source data/Figure 1-source data 3.tif]

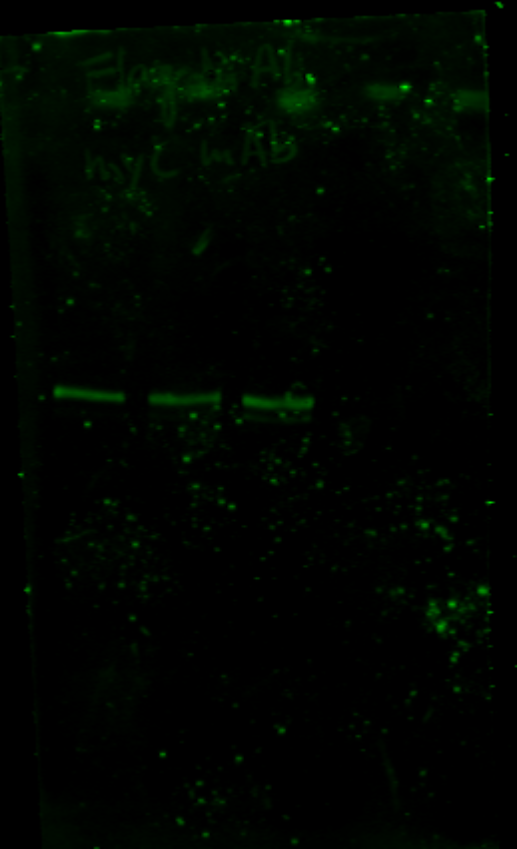

Supplement: Source data 1. [file elife-71142-data1.zip › eLife source data/Figure 1-source data 1.tif]

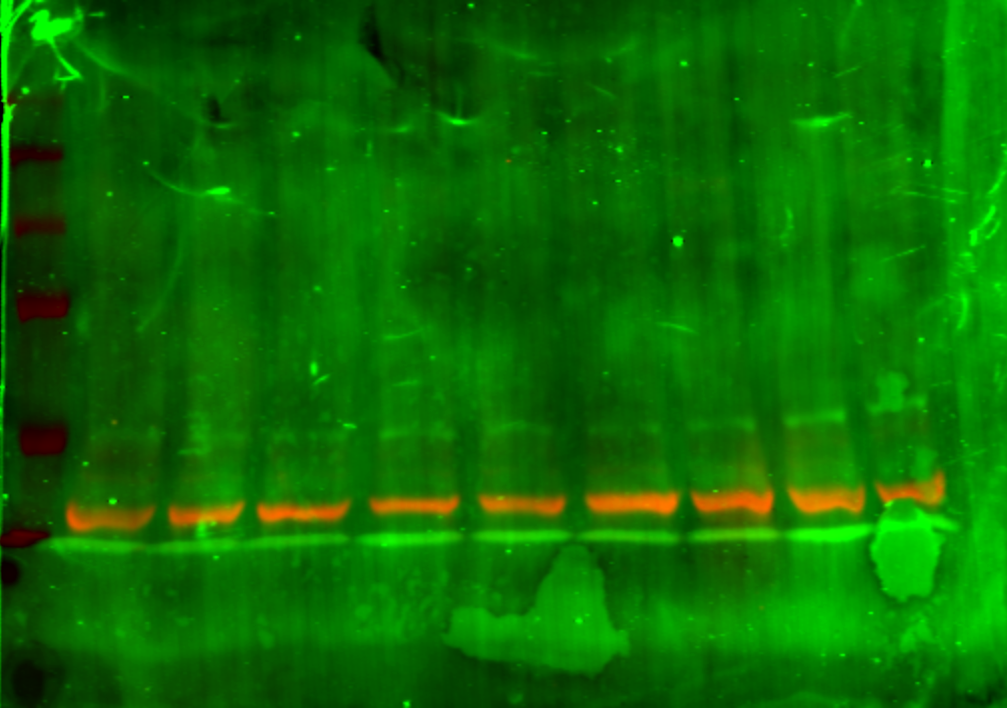

Supplement: Source data 1. [file elife-71142-data1.zip › eLife source data/Figure 9-source data 7.tif]

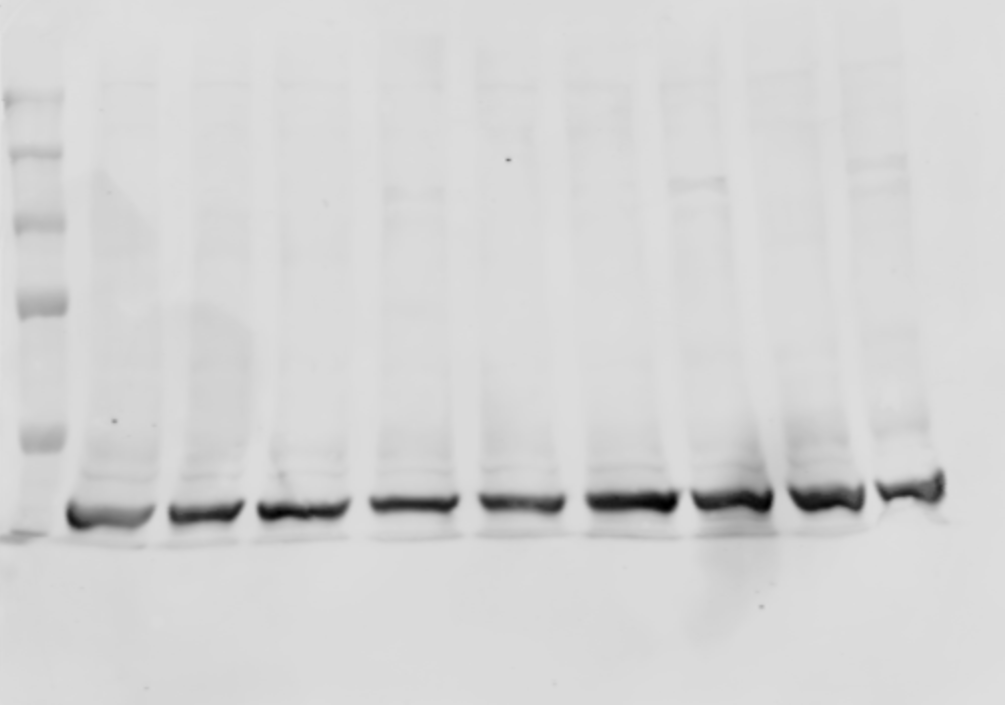

Supplement: Source data 1. [file elife-71142-data1.zip › eLife source data/Figure 9-source data 6.tif]

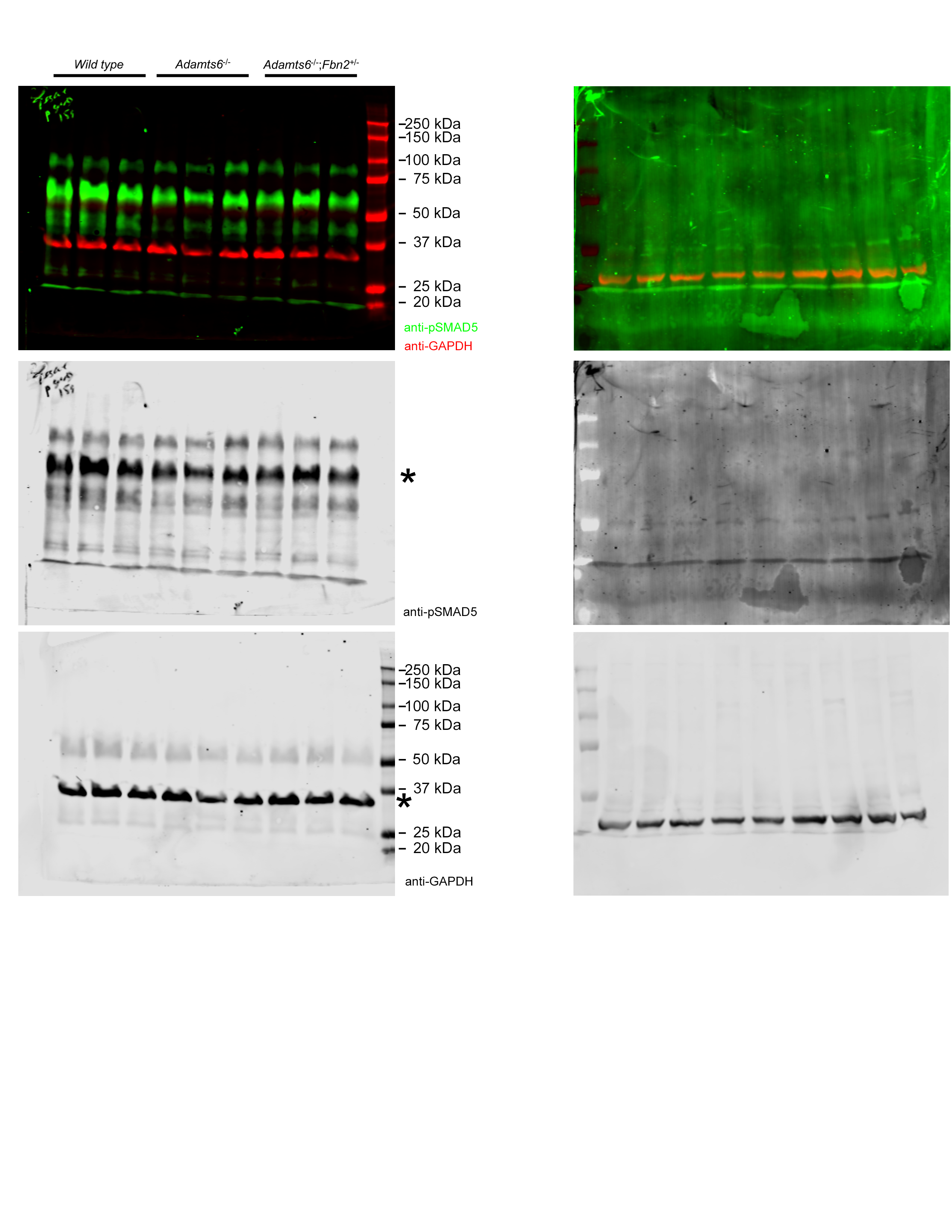

Supplement: Source data 1. [file elife-71142-data1.zip › eLife source data/Figure 9-source data 4.tif]

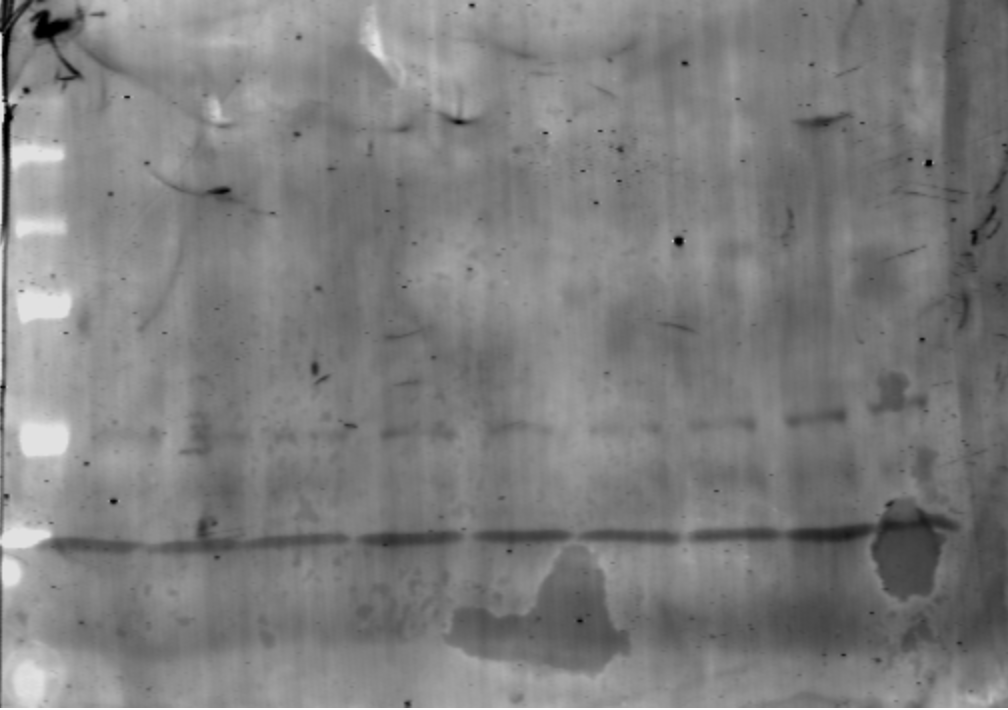

Supplement: Source data 1. [file elife-71142-data1.zip › eLife source data/Figure 9-source data 5.tif]

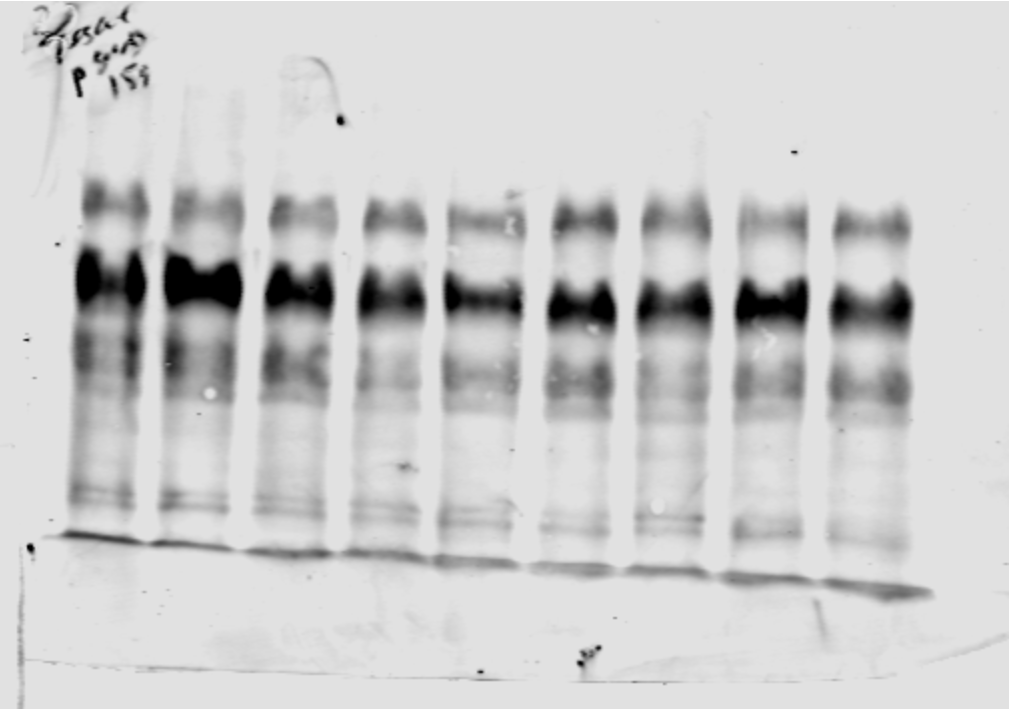

Supplement: Source data 1. [file elife-71142-data1.zip › eLife source data/Figure 9-source data 1.tif]

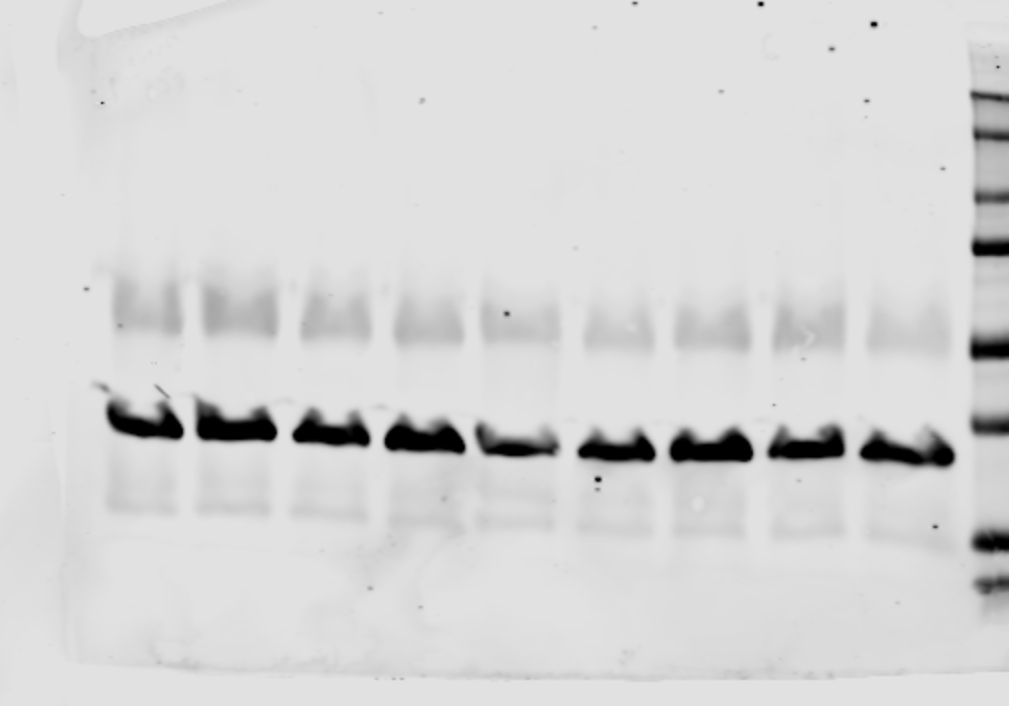

Supplement: Source data 1. [file elife-71142-data1.zip › eLife source data/Figure 9-source data 2.tif]

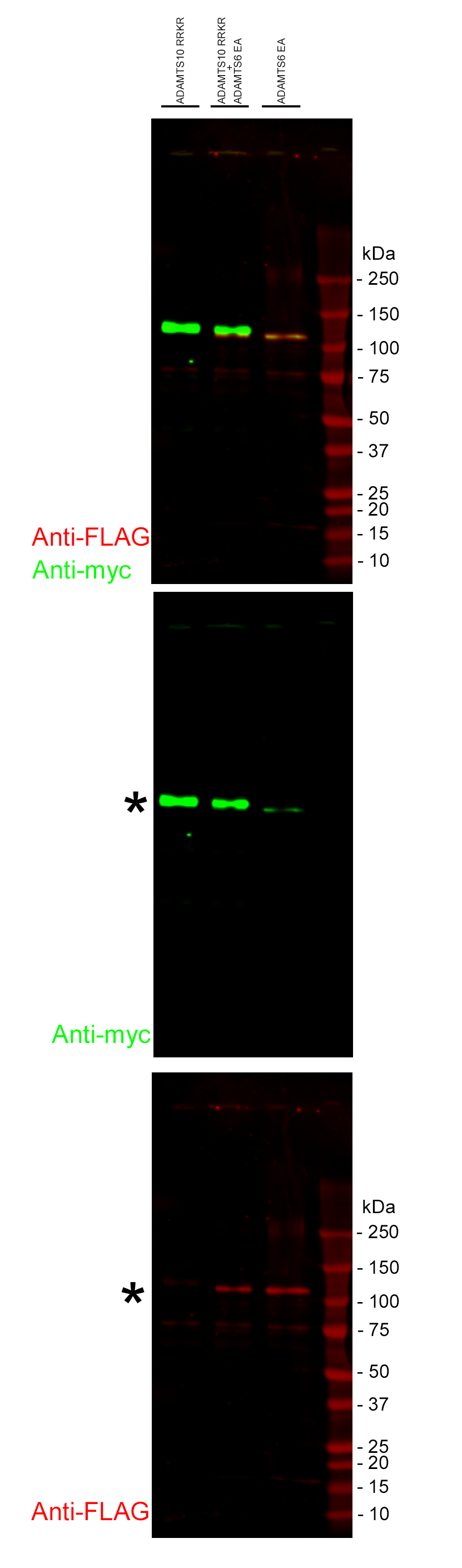

Supplement: Source data 1. [file elife-71142-data1.zip › eLife source data/Figure 1-source data 8.tif]

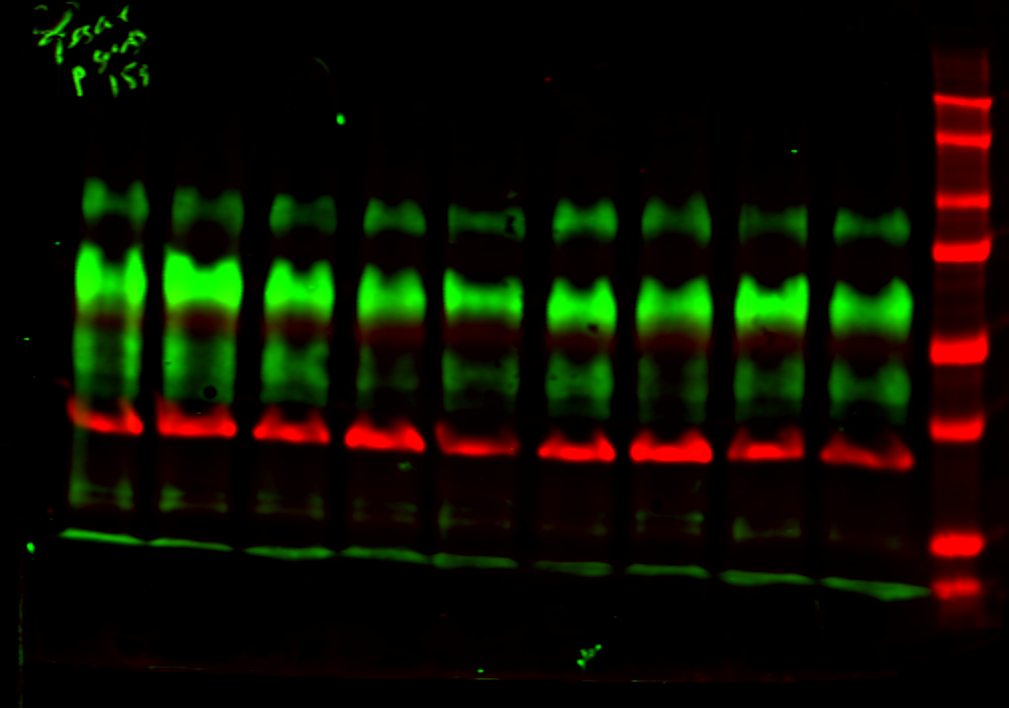

Supplement: Source data 1. [file elife-71142-data1.zip › eLife source data/Figure 9-source data 3.tif]
