# Supplementary material for: Proteolysis of fibrillin-2 microfibrils is essential for normal skeletal development
Source: eLife. 2022 May 3;11:e71142. doi: 10.7554/eLife.71142 (PMC9064305; doi:10.7554/eLife.71142)
Supplement: Supplementary file 1. [file elife-71142-supp1.docx]

**Supplemental Table 1. Antibodies**

| **Name** | **Source** | **Dilution** |
| --- | --- | --- |
| **anti-Fibrillin-2-gly** | (93) | IF: 1:300 |
| **anti-mFbn1-C** | (94) | IF: 1:500 |
| **Anti-MAGP1** | (95) | IF: 1:200 |
| **anti-Sox9** | Millipore AB5535 | IF: 1:300 |
| **anti-Acan** | Millipore AB1031 | IF: 1:400 |
| **anti-CLP** | DHSB 9/30/8-A4-c | IF: 1:100 |
| **anti-Col X** | Abcam ab58632 | IF: 1:1000 |
| **Anti-PCNA** | Cell Signaling 2586S | IF: 1:200 |
| **anti-rFBN2-C** | (21) | WB: 1:500 |
| **anti-His** | R&D MAB050 | IF: 1:400  WB: 1:1000 |
| **anti-pSmad5** | Abcam ab92698 | WB: 1:1000 |
| **anti-pSmad1/5** | Cell Signaling 9516 | IF: 1:200 |
| **anti-pSmad2** | Cell Signaling 3108 | WB: 1:1000  IF: 1:200 |
| **Anti-GDF5** | R&D AF853 | IF: 1:100 |
| **anti-GAPDH** | EMD Millipore MAB374 | WB: 1:5000 |

IF, immunofluorescence; WB, western blot
