# Supplementary material for: Proteolysis of fibrillin-2 microfibrils is essential for normal skeletal development
Source: eLife. 2022 May 3;11:e71142. doi: 10.7554/eLife.71142 (PMC9064305; doi:10.7554/eLife.71142)
Supplement: Supplementary file 2. [file elife-71142-supp2.docx]

**Supplemental Table 2. Quantitative Real-Time PCR primers.**

| *Adamts6* | Forward: 5´-TCTCTAGCTCATAGGTAGCCC-3´ |
| --- | --- |
|  | Reverse: 5´-GTTTAGGTGAAAGTGCTTGCC-3´ |
| *Adamts10* | Forward: 5´-CATCACACGCTATGACATCTG-3´ |
|  | Reverse: 5´-CACGAATGGATTAGTCTTCATGG-3´ |
| *Fbn2* | Forward: 5´-AACGATTGCCTAGACATAGAC-3´ |
|  | Reverse: 5´-TTCGCTTCTCACTTCATATCC-3´ |
| *Fbn1* | Forward: 5´-GCTGTGAATGCGACATGGGCTT-3´ |
|  | Reverse: 5´-TCTCACACTCGCAACGGAAGAG-3´ |
| *Gapdh* | Forward: 5´-TGGAGAAACCTGCCAAGTATGA-3´ |
|  | Reverse: 5´-CTGTTGAAGTCGCAGGAGACA-3´ |
| *Adamts17* | Forward: 5´-TGGCACCAATGCTACCTTCTGC-3´ |
|  | Reverse: 5´-TGTCTGCTCCACATTCAGTGCC-3´ |
| *Adamts19* | Forward: 5´-GGTTTCCTACCACGGAGCAGAT-3´ |
|  | Reverse: 5´-GGAGAATGAGCTTTAGCACACGG-3´ |
